# Supplementary material for: Interaction mapping of endoplasmic reticulum ubiquitin ligases identifies modulators of innate immune signalling
Source: eLife. 2020 Jul 2;9:e57306. doi: 10.7554/eLife.57306 (PMC7332293; doi:10.7554/eLife.57306)
Supplement: Transparent reporting form [file elife-57306-transrepform.docx]

***eLife’s* transparent reporting form (26-03-2020-RA-eLife-57306)**

We encourage authors to provide detailed information *within their submission* to facilitate the interpretation and replication of experiments. Authors can upload supporting documentation to indicate the use of appropriate reporting guidelines for health-related research (see [EQUATOR Network](http://www.equator-network.org/%20)), life science research (see the [BioSharing Information Resource](https://biosharing.org/)), or the [ARRIVE guidelines](http://www.plosbiology.org/article/info:doi/10.1371/journal.pbio.1000412) for reporting work involving animal research. Where applicable, authors should refer to any relevant reporting standards documents in this form.

If you have any questions, please consult our Journal Policies and/or contact us: [editorial@elifesciences.org](mailto:editorial@elifesciences.org).

**Sample-size estimation**

- You should state whether an appropriate sample size was computed when the study was being designed
- You should state the statistical method of sample size computation and any required assumptions
- If no explicit power analysis was used, you should describe how you decided what sample (replicate) size (number) to use

Please outline where this information can be found within the submission (e.g., sections or figure legends), or explain why this information doesn’t apply to your submission:

Sample sizes are indicated in figure legends of each figure (starting page 48), and details of statistical methods are stated in the Materials and Methods (page 34). Each was decided based on prior experience with the assays as well as practical considerations and generally accepted standards in the field. No formal power analysis was calculated as the results were consistent between different samples. Historically, these types of experiments involving the use of cell lines have confidence intervals and standard deviations that are small, providing confidence that they are statistically meaningful.

**Replicates**

- You should report how often each experiment was performed
- You should include a definition of biological versus technical replication
- The data obtained should be provided and sufficient information should be provided to indicate the number of independent biological and/or technical replicates
- If you encountered any outliers, you should describe how these were handled
- Criteria for exclusion/inclusion of data should be clearly stated
- High-throughput sequence data should be uploaded before submission, with a private link for reviewers provided (these are available from both GEO and ArrayExpress)

Please outline where this information can be found within the submission (e.g., sections or figure legends), or explain why this information doesn’t apply to your submission:

For all quantified data (LC-MS/MS, NanoString, WB (main Figure 6B only), and qPCR) the details of number of replicates, analyses, and statistical tests used can be found in methods, figures legends, and extended data table 12.

For all other data the number of replicates and reproducibility of each experiment is described in tables at the end of this document. For convenience, the number of replicates for quantified data are also provided.

**Statistical reporting**

- Statistical analysis methods should be described and justified
- Raw data should be presented in figures whenever informative to do so (typically when N per group is less than 10)
- For each experiment, you should identify the statistical tests used, exact values of N, definitions of center, methods of multiple test correction, and dispersion and precision measures (e.g., mean, median, SD, SEM, confidence intervals; and, for the major substantive results, a measure of effect size (e.g., Pearson's r, Cohen's d)
- Report exact p-values wherever possible alongside the summary statistics and 95% confidence intervals. These should be reported for all key questions and not only when the p-value is less than 0.05.

Please outline where this information can be found within the submission (e.g., sections or figure legends), or explain why this information doesn’t apply to your submission:

Details of analyses and statistical tests used can be found in methods, figures legends, and extended data table 12.

(For large datasets, or papers with a very large number of statistical tests, you may upload a single table file with tests, Ns, etc., with reference to sections in the manuscript.)

**Group allocation**

- Indicate how samples were allocated into experimental groups (in the case of clinical studies, please specify allocation to treatment method); if randomization was used, please also state if restricted randomization was applied
- Indicate if masking was used during group allocation, data collection and/or data analysis

Please outline where this information can be found within the submission (e.g., sections or figure legends), or explain why this information doesn’t apply to your submission:

The nature of our data does not warrant allocation into groups.

**Additional data files (“source data”)**

- We encourage you to upload relevant additional data files, such as numerical data that are represented as a graph in a figure, or as a summary table
- Where provided, these should be in the most useful format, and they can be uploaded as “Source data” files linked to a main figure or table
- Include model definition files including the full list of parameters used
- Include code used for data analysis (e.g., R, MatLab)
- Avoid stating that data files are “available upon request”

Please indicate the figures or tables for which source data files have been provided:

LC-MS/MS source data along with metadata and code used for analysis has been uploaded to the ProteomeXchange PRIDE database. The details for access are provide in the Methods and Materials. Source data for Figures (3B, 6C-E) and figure supplements (Fig 4 – figure supplement 1B, Fig 6 – figure supplement 1A-D) are also included.

| **Quantified data** | | |
| --- | --- | --- |
| **Figure** | **Number of repeats** | **Comments** |
| Main figure 3B | Three biological replicates | - |
| Main figure 6B/C | Three biological replicates | - |
| Main figure 6D | At least four biological replicates | - |
| Main figure 6E | Four biological replicates | - |
| Figure 4 – figure supplement 1B | Three biological replicates | - |
| Figure 6 – figure supplement 1A | Three biological replicates | - |
| Figure 6 – figure supplement 1B | Three biological replicates | - |
| Figure 6 – figure supplement 1C | Four biological replicates | - |
| Figure 6 – figure supplement 1D | At least four biological replicates | - |

| **Non-quantified data** | | |
| --- | --- | --- |
| **Figure** | **Number of repeats** | **Comments** |
| Main figure 1C | Two biological replicates | Independently performed by HA-IP which yielded the same profiles. |
| Main figure 4C | Two biological replicates | - |
| Main figure 4D | Performed once | Validates observations in main figure 4C and F. Confirmed independently for WT RNF26 in main figure 5E. |
| Main figure 4E | Performed once | - |
| Main figure 4F | Two biological replicates | Independently validated in extended data figure 4C. |
| Main figure 5C | Two biological replicates | Validation of LC-MS/MS results from this study. ENDOD1 and TMED1 WBs performed once. |
| Main figure 5D | Two biological replicates | TMEM33, ENDOD1, and TMED1 WBs performed once. |
| Main figure 5E | Two biological replicates | Validation of endogenous interactions found by LC-MS/MS in this study. Knockdowns shown independently in main figure 5F and Figure 5 - figure supplement 1D-G. HA, TMEM43 and TMED1 WBs performed once. |
| Main figure 5F | Performed once | Independently validates observations for WT RNF26 in main figure 4D. Knockdowns shown independently in main figure 5E and Figure 5 - figure supplement 1D-G. |
| Figure 1 -figure supplement 1A | Each WB was performed once | Confirmation of FLAG-HA-tagged E3 expression for each stable cell line generated. FLAG-HA-E3s independently detected by LC-MS/MS in this study. |
| Figure 1 -figure supplement 1B | IF performed once for each cell line | Confirmation that FLAG-HA-tagged E3s reside in the ER (independently shown in Neutzner et al 2011, Maruyama et al 2008, van de Weijer et al 2014) |
| Figure 1 -figure supplement 1C | Performed once | Confirmation of Hrd1 complex assembly shown independently in Schulz et al 2017. |
| Figure 3 -figure supplement 1A | Two biological replicates | - |
| Figure 3 -figure supplement 1C | Performed once | Validation of LC-MS/MS results from this study. |
| Figure 4 -figure supplement 1C | Performed once | Validation of data shown in main figure 4F. |
| Figure 4 -figure supplement 1D | Two biological replicates | - |
| Figure 5 -figure supplement 1B,C | Performed once | Validation of LC-MS/MS results from this study. |
| Figure 5 -figure supplement 1D-G | Each WB was performed once | Knockdowns are shown independently in main figure 5E and F, and also independently confirmed by qPCR (data not shown). |
